# Supplementary material for: Metabolic and inflammatory biomarker trajectories after a cancer diagnosis and the risk of cardiovascular diseases
Source: Nat Commun. 2026 May 25;17:4643. doi: 10.1038/s41467-026-73530-1 (PMC13201860; doi:10.1038/s41467-026-73530-1)
Supplement: Supplementary file 1 — Supplementary Information [file 41467_2026_73530_MOESM1_ESM.pdf]

# Supplementary materials to "Metabolic and inflammatory biomarker trajectories after a cancer diagnosis and the risk of cardiovascular diseases" by Hyemi Park et al.

Supplementary Table 1. Associations between cancer and subsequent risk of cardiovascular disease after excluding patients diagnosed with their first cancer after 1996 (n=635,293) .......................... 2

Supplementary Table 2. Numbers of individuals included in Analysis 1 – The association between cancer and the risk of subsequent cardiovascular diseases .......................................................... 3

Supplementary Table 3. Numbers of individuals included in Analysis 2 - Biomarker trajectories between individuals with and without a history of cancer ............................................................... 4

Supplementary Table 4. Numbers of measurements for different biomarkers included in Analysis 2 - Biomarker trajectories between individuals with and without a history of cancer ............................ 5

Supplementary Table 5. Numbers of individuals included in Analysis 3 – Latent classes of biomarker trajectories following a cancer diagnosis and the associations with subsequent cardiovascular diseases ....................................................................................................................................... 6

Supplementary Table 6. Associations between latent classes of longitudinal biomarker trajectories and the risk of cardiovascular disease following a cancer diagnosis among the three most frequent cancer types by organ system ......................................................................................................... 7

Supplementary Figure 1. Associations between cancer and the risk of cardiovascular disease by the site of cancer ........................................................................................................................... 8

Supplementary Figure 2. Proportion of individuals undergoing biomarker testing according to the time since cancer diagnosis ........................................................................................................ 9

**Supplementary Table 1. Associations between cancer and subsequent risk of cardiovascular disease after excluding patients diagnosed with their first cancer after 1996 (n=635,293)**

| Exposure | Number of study individuals (events) | Rate, per 1000 person-years | Multivariable adjusted IRR (95% CI)* |
|---|---|---|---|
| **Any cancer** | | | |
| No | 611,449/280,623 | 17.0 | |
| Yes | 23,844/12,809 | 52.5 | 1.39 (1.36-1.41) |
| **Age at cancer diagnosis** | | | |
| <18 years | 257/52 | 10.4 | 2.62 (2.00-3.44) |
| 18-44 years | 3713/1550 | 23.3 | 1.56 (1.48-1.64) |
| 45-54 years | 4937/2629 | 40.7 | 1.43 (1.37-1.48) |
| 55-64 years | 6269/3508 | 60.7 | 1.33 (1.28-1.37) |
| >64 years | 8668/5070 | 101.1 | 1.35 (1.31-1.39) |

IRR, incidence rate ratio; CI, confidence intervals.

*Adjusted for age and calendar period of follow-up, sex, year of birth, country of birth, income, education, and employment status at the first blood sampling, as well as diabetes and psychiatric disorders by the end of follow-up.

**Supplementary Table 2. Numbers of individuals included in Analysis 1 – The association between cancer and the risk of subsequent cardiovascular diseases**

| Cancer by organ systems | Number of cancer patients during follow-up | Cancer by sites | Number of cancer patients during follow-up |
|---|---|---|---|
| Breast and reproductive system | 41,306 | Breast | 16,334 |
| Digestive system | 13,603 | Colon | 5172 |
| Hematological malignancy | 6784 | Melanoma | 5073 |
| Respiratory system | 5552 | Lung | 5041 |
| Urinary system | 5165 | Non-melanoma skin | 3951 |
| Central nervous system | 2630 | Non-Hodgkin's lymphoma | 3646 |
| Buccal cavity and pharynx | 1652 | Rectum | 3474 |
| Others | 17,062 | Uterus | 2767 |
| | | Central nervous system | 2630 |
| | | Kidney | 1650 |
| | | Other endocrine gland | 1643 |
| | | Ovary | 1561 |
| | | Pancreas | 1504 |
| | | Stomach | 1270 |
| | | Cervix | 1153 |
| | | Liver | 1044 |
| | | Testis | 892 |
| | | Acute myeloid leukemia | 742 |
| | | Thyroid | 693 |
| | | Esophagus | 601 |
| | | Connective tissue | 574 |
| | | Small intestine | 369 |
| | | Hodgkin's lymphoma | 352 |
| | | Eye | 237 |
| | | Salivary gland | 185 |
| | | Acute lymphoid leukemia | 165 |
| | | Bone | 139 |
| | | Other or unspecified sites | 27,892 |

**Supplementary Table 3. Numbers of individuals included in Analysis 2 - Biomarker trajectories between individuals with and without a history of cancer**

| Biomarkers | Total | Non-cancer individuals | Cancer patients | Cancer by organ systems | | | | | | | |
|---|---|---|---|---|---|---|---|---|---|---|---|
| | | | | Breast and reproductive system | Digestive system | Urinary system | Hematological malignancy | Central nervous system | Respiratory system | Buccal cavity and pharynx | Others |
| **Glucose** | 12,314 | 10,522 | 1792 | 804 | 214 | 141 | 79 | 51 | 37 | 37 | 429 |
| **Fructosamine** | 11,539 | 9948 | 1591 | 716 | 191 | 128 | 72 | 47 | 32 | 31 | 374 |
| **TC** | 13,257 | 11,344 | 1913 | 876 | 212 | 149 | 78 | 55 | 42 | 38 | 463 |
| **HDL** | 4775 | 4087 | 688 | 303 | 74 | 54 | 30 | 18 | 16 | 16 | 177 |
| **LDL** | 4999 | 4281 | 718 | 314 | 77 | 57 | 32 | 20 | 19 | 16 | 183 |
| **LDL/HDL ratio** | 4773 | 4089 | 684 | 297 | 71 | 56 | 30 | 18 | 17 | 16 | 179 |
| **TG** | 13,202 | 11,304 | 1898 | 862 | 211 | 152 | 77 | 54 | 42 | 38 | 462 |
| **ApoA1** | 5064 | 4337 | 727 | 316 | 78 | 59 | 31 | 20 | 20 | 16 | 187 |
| **ApoB** | 5361 | 4636 | 725 | 321 | 74 | 61 | 29 | 21 | 19 | 15 | 185 |
| **ApoB/ApoA1 ratio** | 5450 | 4745 | 705 | 309 | 73 | 56 | 29 | 21 | 19 | 15 | 183 |
| **Leukocyte** | 10,243 | 8869 | 1374 | 629 | 187 | 107 | 98 | 25 | 32 | 19 | 277 |
| **Albumin** | 14,334 | 12,273 | 2061 | 923 | 265 | 171 | 84 | 56 | 45 | 35 | 482 |
| **IgG** | 631 | 476 | 155 | 63 | 18 | 14 | 12 | 5 | 3 | NA | 40 |
| **CRP** | 11,987 | 10,466 | 1521 | 691 | 176 | 132 | 65 | 40 | 34 | 31 | 352 |
| **Haptoglobin** | 7203 | 6167 | 1036 | 456 | 110 | 79 | 55 | 30 | 20 | 24 | 262 |
| **Uric acid** | 12,283 | 10,446 | 1837 | 829 | 213 | 150 | 77 | 54 | 39 | 35 | 440 |

TC, total cholesterol; HDL, high-density lipoprotein; LDL, low-density lipoprotein cholesterol; TG, triglycerides; ApoA1, apolipoprotein A1; ApoB, Apolipoprotein B; IgG, immunoglobulin G; CRP, C-reactive protein.

**Supplementary Table 4. Numbers of measurements for different biomarkers included in Analysis 2 - Biomarker trajectories between individuals with and without a history of cancer**

| Biomarker | Number of tests, median (IQR) | |
|---|---|---|
| | Individuals with cancer | Individuals without cancer |
| Glucose | 4 (3-6) | 3 (2-4) |
| Fructosamine | 4 (3-6) | 3 (2-4) |
| TC | 4 (3-6) | 3 (2-4) |
| HDL | 3 (2-5) | 2 (2-4) |
| LDL | 3 (2-5) | 2 (2-4) |
| LDL/HDL ratio | 3 (2-5) | 2 (2-4) |
| TG | 4 (3-6) | 3 (2-4) |
| ApoA1 | 3 (2-5) | 2 (2-4) |
| ApoB | 3 (2-5) | 2 (2-4) |
| ApoB/ApoA1 ratio | 3 (2-5) | 2 (2-4) |
| Leukocyte | 3 (3-6) | 2 (2-3) |
| Albumin | 3 (3-5) | 3 (2-4) |
| IgG | 2 (2-3) | 2 (2-3) |
| CRP | 3 (2-5) | 3 (2-4) |
| Haptoglobin | 3 (2-4) | 3 (2-4) |
| Uric acid | 3 (3-6) | 3 (2-4) |

TC, total cholesterol; HDL, high-density lipoprotein; LDL, low-density lipoprotein cholesterol; TG, triglycerides; ApoA1, apolipoprotein A1; ApoB, Apolipoprotein B; IgG, immunoglobulin G; CRP, C-reactive protein.

**Supplementary Table 5. Numbers of individuals included in Analysis 3 – Latent classes of biomarker trajectories following a cancer diagnosis and the associations with subsequent cardiovascular diseases**

| Biomarkers | Total | Cancer by organ systems | | | | | | | |
|---|---|---|---|---|---|---|---|---|---|
| | | Breast and reproductive system | Digestive system | Urinary system | Hematological malignancy | Central nervous system | Respiratory system | Buccal cavity and pharynx | Others |
| **Glucose** | 1460 | 660 | 167 | 108 | 66 | 44 | 31 | 32 | 352 |
| **TC** | 1577 | 719 | 168 | 116 | 66 | 49 | 33 | 31 | 395 |
| **HDL** | 426 | 201 | 43 | 26 | 20 | 8 | 10 | 11 | 107 |
| **LDL** | 442 | 205 | 45 | 28 | 21 | 9 | 11 | 11 | 112 |
| **LDL/HDL ratio** | 426 | 201 | 43 | 26 | 20 | 8 | 10 | 11 | 107 |
| **ApoA1** | 448 | 207 | 47 | 29 | 21 | 9 | 11 | 11 | 113 |
| **ApoB** | 455 | 212 | 45 | 30 | 21 | 11 | 12 | 10 | 114 |
| **ApoB/ApoA1 ratio** | 428 | 200 | 43 | 29 | 20 | 9 | 11 | 10 | 106 |
| **Albumin** | 1584 | 711 | 195 | 124 | 63 | 46 | 32 | 32 | 381 |
| **CRP** | 1038 | 471 | 123 | 79 | 42 | 29 | 22 | 19 | 253 |
| **Uric acid** | 1439 | 651 | 166 | 111 | 61 | 44 | 32 | 30 | 344 |

TC, total cholesterol; HDL, high-density lipoprotein; LDL, low-density lipoprotein cholesterol; ApoA1, apolipoprotein A1; ApoB, Apolipoprotein B; CRP, C-reactive protein.

**Supplementary Table 6. Associations between latent classes of longitudinal biomarker trajectories and the risk of cardiovascular disease following a cancer diagnosis among the three most frequent cancer types by organ system**

| Biomarkers | Latent classes | Hazard ratio (95% confidence intervals)† | | |
|---|---|---|---|---|
| | | Breast and reproductive system | Digestive system | Urinary system |
| Glucose | 1: stable at low levels | Ref. | Ref. | Ref. |
| | 2: started at high levels and increased | 2.37 (1.55-3.6) | 2.83 (1.18-6.78) | 3.11 (1.13-8.56) |
| TC | 1: started low with a U-shape pattern | 0.83 (0.56-1.22) | 0.65 (0.22-1.91) | 1.15 (0.53-2.53) |
| | 2: stable at high levels | Ref. | Ref. | Ref. |
| Albumin | 1: low-stable levels | 0.47 (0.33-0.68) | 0.50 (0.23-1.10) | 0.85 (0.38-1.86) |
| | 2: moderate-stable levels | Ref. | Ref. | Ref. |
| | 3: high-stable levels | 0.41 (0.28-0.59) | 0.87 (0.39-1.93) | 0.88 (0.39-1.99) |
| CRP | 1: started at low levels, rapidly increased, then decreased | 1.03 (0.61-1.77) | 1.04 (0.82-1.33) | 0.67 (0.35-1.27) |
| | 2: started at high levels, slightly decreased, then increased | Ref. | Ref. | Ref. |
| Uric acid | 1: started at low levels, remained stable, then increased | 1.22 (0.75-1.96) | 1.20 (0.98-1.46) | 1.22 (0.54-2.76) |
| | 2: started at high levels and remained relatively stable | Ref. | Ref. | Ref. |

TC, total cholesterol; CRP, C-reactive protein.
†Attained age was used as the time scale in the Cox regression models. Multivariable models adjusted for sex, year at cancer diagnosis, country of birth, education, income, and employment status at the first blood sampling, and psychiatric disorders and diabetes by the end of follow-up.

| Cancer by sites | No. events | Rate* | Multivariable model** IRR (95%CI) |
|---|---|---|---|
| Pancreas | 402 | 305.4 | 6.55 (5.94–7.22) |
| Liver | 339 | 219.4 | 4.64 (4.17–5.17) |
| Esophagus | 218 | 236.5 | 4.58 (4.01–5.23) |
| Lung | 1820 | 183.6 | 4.09 (3.91–4.28) |
| Acute lymphoid leukemia | 55 | 31.9 | 3.90 (3.00–5.08) |
| Other or unspecified sites | 442 | 130.0 | 3.03 (2.76–3.32) |
| Acute myeloid leukemia | 305 | 103.3 | 2.42 (2.17–2.71) |
| Small intestine | 191 | 94.2 | 2.38 (2.07–2.75) |
| Hodgkin's lymphoma | 171 | 40.6 | 2.32 (2.00–2.69) |
| Bone | 54 | 45.0 | 2.06 (1.58–2.69) |
| Stomach | 436 | 103.8 | 2.00 (1.82–2.20) |
| Central Nervous system | 1169 | 58.0 | 1.95 (1.84–2.07) |
| Kidney | 866 | 83.9 | 1.89 (1.77–2.02) |
| Rectum | 1929 | 101.0 | 1.85 (1.77–1.94) |
| Ovary | 634 | 61.6 | 1.82 (1.69–1.97) |
| Cervix | 498 | 40.8 | 1.74 (1.60–1.90) |
| Non−Hodgkin's lymphoma | 1878 | 76.4 | 1.71 (1.63–1.79) |
| Salivary gland | 85 | 55.6 | 1.67 (1.35–2.06) |
| Colon | 2610 | 89.4 | 1.64 (1.57–1.70) |
| Breast | 8645 | 59.1 | 1.56 (1.52–1.59) |
| Connective tissue | 270 | 54.8 | 1.50 (1.33–1.69) |
| Otherocrine gland | 934 | 50.6 | 1.46 (1.37–1.56) |
| Uterus | 1678 | 71.9 | 1.44 (1.37–1.51) |
| Thyroid | 292 | 34.8 | 1.32 (1.18–1.48) |
| Melanoma | 2401 | 47.1 | 1.29 (1.24–1.34) |
| Testis | 367 | 23.4 | 1.29 (1.17–1.43) |
| Eye | 91 | 52.8 | 1.23 (1.00–1.51) |
| Non−melanoma Skin | 2179 | 78.4 | 1.14 (1.09–1.19) |

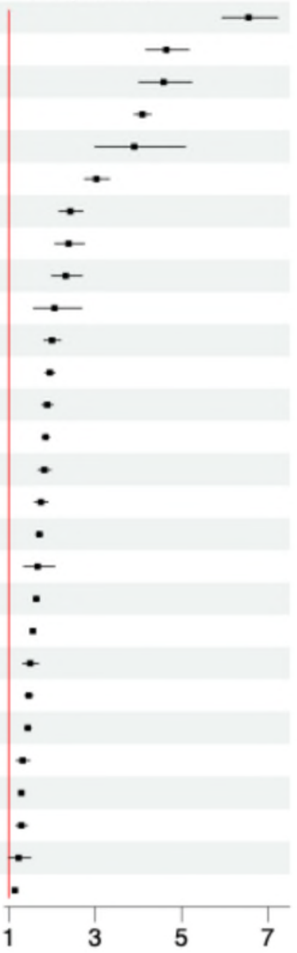


**Supplementary Figure 1. Associations between cancer and the risk of cardiovascular disease by the site of cancer**

∗per 1000 person-years

∗∗Adjusted for age and calendar period of follow-up, sex, year of birth, country of birth, income, education, and employment status at the first blood sampling, as well as diabetes and psychiatric disorders during follow-up.

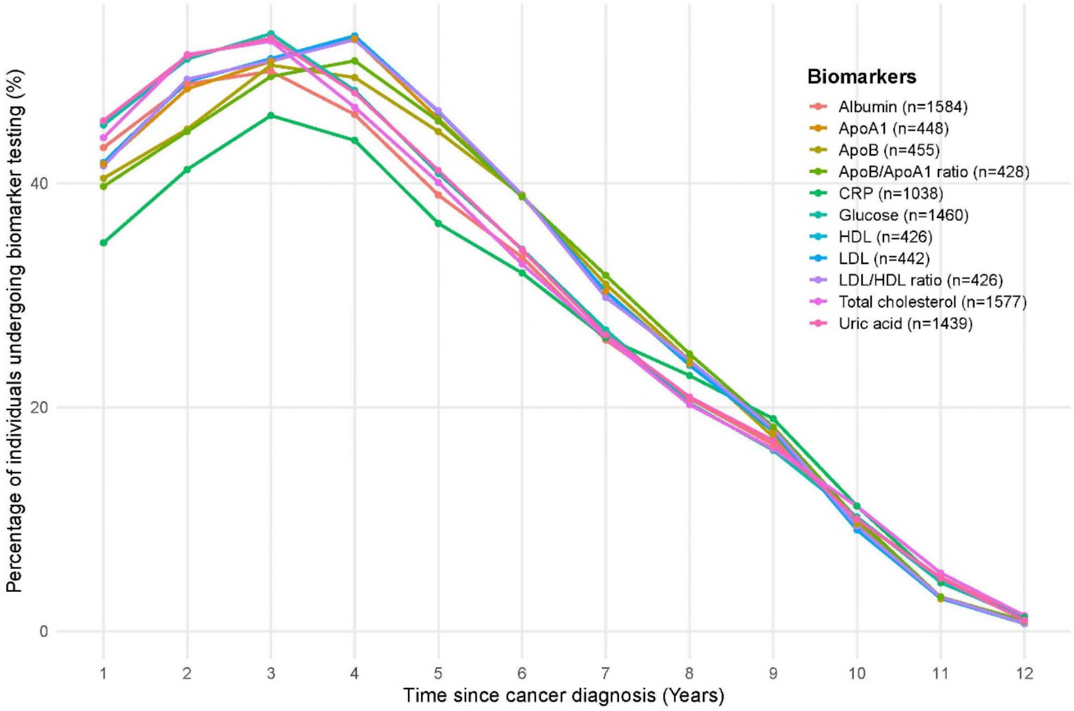


**Supplementary Figure 2. Proportion of individuals undergoing biomarker testing according to the time since cancer diagnosis**
