# Supplementary material for: Metabolic and inflammatory biomarker trajectories after a cancer diagnosis and the risk of cardiovascular diseases
Source: Nat Commun. 2026 May 25;17:4643. doi: 10.1038/s41467-026-73530-1 (PMC13201860; doi:10.1038/s41467-026-73530-1)
Supplement: Supplementary file 2 — Description of Additional Supplementary Files [file 41467_2026_73530_MOESM2_ESM.pdf]

Supplementary Datasets

Supplementary Dataset 1. Characteristics of individuals from AMORIS included in the present study
SD, standard deviation.

Supplementary Dataset 2. Parameters of biomarker trajectories from latent class growth modelling
C indicates number of classes; TC, total cholesterol; HDL, high-density lipoprotein; LDL, low-density lipoprotein cholesterol; TG, triglycerides; ApoA1, apolipoprotein AI; ApoB, Apolipoprotein B; CRP, C-reactive protein. The selected model is represented as bold. * Failed to converge; ** There was no best fit model for fructosamine, triglycerides, leukocyte, and haptoglobin according to the criteria of model selection. a Linear, b Quadratic.

Supplementary Dataset 3. Associations between latent classes of longitudinal biomarker trajectories and the risk of cardiovascular disease following a cancer diagnosis
TC, total cholesterol; HDL, high-density lipoprotein; LDL, low-density lipoprotein cholesterol; TG, triglycerides; ApoA1, Apolipoprotein A1; ApoB, Apolipoprotein B; IgG, immunoglobulin G; CRP, C-reactive protein.
The groups with the largest number of individuals were assigned as the reference group.
*per 1000 person-years.
†Attained age was used as the time scale in the Cox regression models. Multivariable models adjusted for sex, year at cancer diagnosis, country of birth, education, income, and employment status at the first blood sampling, and psychiatric disorders and diabetes by the end of follow-up.

Supplementary Dataset 4. Associations between latent classes of biomarker trajectories and the risk of subtypes of cardiovascular disease following a cancer diagnosis
TC, total cholesterol; HDL, high-density lipoprotein; LDL, low-density lipoprotein cholesterol; ApoA1, apolipoprotein AI; ApoB, Apolipoprotein B; CRP, C-reactive protein.
∗Adjusted for sex, year at cancer diagnosis, country of birth, education, income, and employment status at the first blood sampling, and psychiatric disorders and diabetes by the end of follow-up.

Supplementary Dataset 5. International Classification of Diseases (ICD) codes used in the present study

Supplementary Dataset 6. Comparison of characteristics among individuals with cancer diagnosis during 1985-1996 in the AMORIS cohort according to the number of biomarker measurements
SD, standard deviation.
