# Supplementary material for: Metabolic and inflammatory biomarker trajectories after a cancer diagnosis and the risk of cardiovascular diseases
Source: Nat Commun. 2026 May 25;17:4643. doi: 10.1038/s41467-026-73530-1 (PMC13201860; doi:10.1038/s41467-026-73530-1)
Supplement: Supplementary file 4 — Reporting Summary [file 41467_2026_73530_MOESM4_ESM.pdf]

nature portfolio

Corresponding author(s): Kejia Hu

Last updated by author(s): Oct 12, 2025

Reporting Summary

Nature Portfolio wishes to improve the reproducibility of the work that we publish. This form provides structure for consistency and transparency in reporting. For further information on Nature Portfolio policies, see our Editorial Policies and the Editorial Policy Checklist.

Statistics

For all statistical analyses, confirm that the following items are present in the figure legend, table legend, main text, or Methods section.

n/a	Confirmed	
☐	☒	The exact sample size (*n*) for each experimental group/condition, given as a discrete number and unit of measurement
☐	☒	A statement on whether measurements were taken from distinct samples or whether the same sample was measured repeatedly
☐	☒	The statistical test(s) used AND whether they are one- or two-sided<br>*Only common tests should be described solely by name; describe more complex techniques in the Methods section.*
☐	☒	A description of all covariates tested
☐	☒	A description of any assumptions or corrections, such as tests of normality and adjustment for multiple comparisons
☐	☒	A full description of the statistical parameters including central tendency (e.g. means) or other basic estimates (e.g. regression coefficient) AND variation (e.g. standard deviation) or associated estimates of uncertainty (e.g. confidence intervals)
☐	☒	For null hypothesis testing, the test statistic (e.g. *F*, *t*, *r*) with confidence intervals, effect sizes, degrees of freedom and *P* value noted<br>*Give P values as exact values whenever suitable.*
☒	☐	For Bayesian analysis, information on the choice of priors and Markov chain Monte Carlo settings
☐	☒	For hierarchical and complex designs, identification of the appropriate level for tests and full reporting of outcomes
☒	☐	Estimates of effect sizes (e.g. Cohen's *d*, Pearson's *r*), indicating how they were calculated

Our web collection on statistics for biologists contains articles on many of the points above.

Software and code

Policy information about availability of computer code

Data collection	All data management was performed using SAS version 9.4 and R version 4.5.0.
Data analysis	All analyses were performed using SAS version 9.4 and R version 4.5.0. The codes used for analyses in this study are available at Supplementary Information.

For manuscripts utilizing custom algorithms or software that are central to the research but not yet described in published literature, software must be made available to editors and reviewers. We strongly encourage code deposition in a community repository (e.g. GitHub). See the Nature Portfolio guidelines for submitting code & software for further information.

Data

Policy information about availability of data

All manuscripts must include a data availability statement. This statement should provide the following information, where applicable:

- Accession codes, unique identifiers, or web links for publicly available datasets
- A description of any restrictions on data availability
- For clinical datasets or third party data, please ensure that the statement adheres to our policy

The data used in this study can be made available upon request to the Research Data Office at Karolinska Institutet via rdo@ki.se after ensuring compliance with relevant legislation and General Data Protection Regulation. Source data for figures 2-5, Supplementary figures 1-2 and disaggregated data of sex were provided with this paper.

Research involving human participants, their data, or biological material

Policy information about studies with human participants or human data. See also policy information about sex, gender (identity/presentation), and sexual orientation and race, ethnicity and racism.

Reporting on sex and gender	We have used the term sex throughout the manuscript to represent biological attribute as this is the only information available from the Swedish Total Population Register. Sex was considered in the study design, and sex of participants was determined based on assigned. We have reported disaggregated for sex in Source data 1. In Analysis 1, there were 757,040 individuals [371,286 (49.0%) males, 385,754 (51.0%) females]. In Analysis 2, we included 13,437 (39.8%) males and 20,323 (60.2%) females. In analysis, we included 2206 individuals [901 (40.8%) males, 1305 (59.2%) females]. We however did not design sex- and gender-based analyses due to sample size.
Reporting on race, ethnicity, or other socially relevant groupings	We did not report race or ethnicity as these variables are not available in Swedish Register data. We have country of birth, income, education, and employment status at the first blood sampling as covariates to represent socioeconomic status.
Population characteristics	757,040 were included in the current study. The mean age at study entry is 35.5 (standard error 17.4), 3069 (0.4%) have a diagnosis of diabetes before 1985 and 19,517 (2.6%) have a diagnosis of psychiatric disorder before 1985.
Recruitment	The AMORIS cohort includes 812,073 Swedish residents with laboratory analyses of blood or urine samples collected for outpatient visits or regular health check-ups in the occupational setting in Stockholm during 1985-1996 (i.e., inclusion period).
Ethics oversight	This study was approved by the Swedish Ethical Review Authority (2020-01545).

Note that full information on the approval of the study protocol must also be provided in the manuscript.

Field-specific reporting

Please select the one below that is the best fit for your research. If you are not sure, read the appropriate sections before making your selection.

☒ Life sciences ☐ Behavioural & social sciences ☐ Ecological, evolutionary & environmental sciences

For a reference copy of the document with all sections, see nature.com/documents/nr-reporting-summary-flat.pdf

Life sciences study design

All studies must disclose on these points even when the disclosure is negative.

Sample size	No sample size was calculated before the analysis as we have used all the available data in all analyses. In Analysis 1, We conducted a cohort study to evaluate the association between cancer and the risk of CVD including a total of 757,040 individuals who were free of cancer and CVD before 1985 in the AMORIS cohort. In Analysis 2, We conducted a matched cohort study per biomarker, including patients diagnosed with the first cancer during 1985-1996 and cancer-free persons individually matched by a propensity score. In Analysis 3, We conducted a cohort study per biomarker among cancer patients who had no prior CVD but at least three measurements of the biomarker after cancer diagnosis, leading to 2206 individuals included in the analysis. All details of the numbers in each analysis is available in Figure 1.
Data exclusions	We have excluded 55,033 individuals:<br>• 5745 with no linkage to TPR<br>• 6034 with reused or changed ID numbers<br>• 10,119 with cancer before study entry<br>• 33,135 with CVD before study entry
Replication	We have ensured reproducibility by describing all steps of study design, experiment and analysis. Attempts at replication were successful.
Randomization	Randomization is not relevant to the current study as there is no intervention to the participants.
Blinding	Blinding is not relevant to the current study as there is no intervention to the participants.

Reporting for specific materials, systems and methods

We require information from authors about some types of materials, experimental systems and methods used in many studies. Here, indicate whether each material, system or method listed is relevant to your study. If you are not sure if a list item applies to your research, read the appropriate section before selecting a response.

Materials & experimental systems

n/a	Involved in the study
☒	☐ Antibodies
☒	☐ Eukaryotic cell lines
☒	☐ Palaeontology and archaeology
☒	☐ Animals and other organisms
☒	☐ Clinical data
☒	☐ Dual use research of concern
☒	☐ Plants

Methods

n/a	Involved in the study
☒	☐ ChIP-seq
☒	☐ Flow cytometry
☒	☐ MRI-based neuroimaging

Plants

Seed stocks	*Report on the source of all seed stocks or other plant material used. If applicable, state the seed stock centre and catalogue number. If plant specimens were collected from the field, describe the collection location, date and sampling procedures.*
Novel plant genotypes	*Describe the methods by which all novel plant genotypes were produced. This includes those generated by transgenic approaches, gene editing, chemical/radiation-based mutagenesis and hybridization. For transgenic lines, describe the transformation method, the number of independent lines analyzed and the generation upon which experiments were performed. For gene-edited lines, describe the editor used, the endogenous sequence targeted for editing, the targeting guide RNA sequence (if applicable) and how the editor was applied.*
Authentication	*Describe any authentication procedures for each seed stock used or novel genotype generated. Describe any experiments used to assess the effect of a mutation and, where applicable, how potential secondary effects (e.g. second site T-DNA insertions, mosiacism, off-target gene editing) were examined.*
